# Evolution of the analytical scattering model of live *Escherichia coli*

**DOI:** 10.1107/S1600576721000169

**Published:** 2021-03-03

**Authors:** Enrico F. Semeraro, Lisa Marx, Johannes Mandl, Moritz P. K. Frewein, Haden L. Scott, Sylvain Prévost, Helmut Bergler, Karl Lohner, Georg Pabst

**Affiliations:** a University of Graz, Institute of Molecular Biosciences, NAWI Graz, 8010 Graz, Austria; b BioTechMed Graz, 8010 Graz, Austria; c Field of Excellence BioHealth – University of Graz, Graz, Austria; d Institut Laue–Langevin, 38043 Grenoble, France; e University of Tennessee, Center for Environmental Biotechnology, Knoxville, Tennessee, USA

**Keywords:** bacterial ultrastructure, small-angle scattering, ultra-small-angle X-ray scattering, USAXS, very small angle neutron scattering, VSANS, compositional modeling

## Abstract

A previously reported multi-scale model for (ultra-)small-angle X-ray (USAXS/SAXS) and (very) small-angle neutron scattering (VSANS/SANS) of live *Escherichia coli* was revised on the basis of compositional/metabolomic and ultrastructural constraints. The cellular body is modeled, as previously described, by an ellipsoid with multiple shells. However, scattering originating from flagella was replaced by a term accounting for the oligosaccharide cores of the lipopolysaccharide leaflet of the outer membrane including its cross-term with the cellular body. This was mainly motivated by (U)SAXS experiments showing indistinguishable scattering for bacteria in the presence and absence of flagella or fimbrae. The revised model succeeded in fitting USAXS/SAXS and differently contrasted VSANS/SANS data of *E. coli* ATCC 25922 over four orders of magnitude in length scale. Specifically, this approach provides detailed insight into structural features of the cellular envelope, including the distance of the inner and outer membranes, as well as the scattering length densities of all bacterial compartments. The model was also successfully applied to *E. coli* K12, used for the authors’ original modeling, as well as for two other *E. coli* strains. Significant differences were detected between the different strains in terms of bacterial size, intermembrane distance and its positional fluctuations. These findings corroborate the general applicability of the approach outlined here to quantitatively study the effect of bactericidal compounds on ultrastructural features of Gram-negative bacteria without the need to resort to any invasive staining or labeling agents.

## Abstract 2

Structural and compositional information about *Escherichia coli* cells is summarized and translated into an analytical multi-length-scale scattering form factor model of live bacterial suspensions.

## 1. Introduction   

*Escherichia coli* is among the most studied Gram-negative bacterial strains in life sciences, with numerous reports on its structure and composition (Breed & Dotterrer, 1916 ▸; Lieb *et al.*, 1955 ▸; Maclean & Munson, 1961 ▸; Neidhardt *et al.*, 1990 ▸; Seltmann & Holst, 2002 ▸; Silhavy *et al.*, 2010 ▸). Transmission electron microscopy (TEM) has been an indispensable tool to derive the cell’s ultrastructure, *i.e.* the few tens of nanometres thick structure of the bacterial cell wall (Milne & Subramaniam, 2009 ▸). It took, however, significant efforts to minimize limitations originating from the invasive nature of the technique (Hobot *et al.*, 1984 ▸; Matias *et al.*, 2003 ▸). Moreover, TEM does not allow dynamic studies of the cellular ultrastructure under physiological relevant conditions and thus real-time insight on the modification of *E. coli* by bactericidal compounds, such as antimicrobial peptides. Time-resolved small-angle scattering experiments (Huxley *et al.*, 1980 ▸), capable of probing structural heterogeneities on the (sub)micrometre to subnanometre length scales without the need of using either bulky labels or invasive staining techniques, are possible solutions for this issue [see *e.g.* Zemb & Lindner (2002 ▸) for an overview on scattering techniques]. In terms of static, equilibrium experiments, small-angle neutron scattering (SANS) has been used, for example, to probe the response of thylakoid membranes in live chloroplasts to external stimuli (Liberton *et al.*, 2013 ▸; Nagy *et al.*, 2014 ▸), while a principle component analysis of small-angle X-ray scattering (SAXS) data was applied to get some qualitative insight on the effect of antibiotics on *E. coli* (von Gundlach, Garamus, Gorniak *et al.*, 2016 ▸; von Gundlach, Garamus, Willey *et al.*, 2016 ▸).

Obtaining quantitative insight on a live cell’s ultrastructure using either SAXS or SANS is challenging, however. This is simply because both techniques provide a global average of the entire cellular content, making it very difficult to single out individual contributors (Semeraro *et al.*, 2020 ▸). This can be addressed with extensive use of the contrast variation capabilities of SANS. For example Nickels *et al.* (2017 ▸) were able to grow fully deuterated *Bacillus subtilis* fed with mixtures of protiated and deuterated fatty acids, which allowed them to highlight nanoscopic domains within the bacteria’s cytoplasmic membrane using SANS. Yet, it is also possible to obtain insight on cellular ultrastructure without the need to grow bacteria in D_2_O. In particular, we recently reported a multi-scale model that successfully describes the scattered intensities of live *E. coli* K12 originating from ultra-SAXS (USAXS), SAXS and SANS experiments at five D_2_O/H_2_O ratios (Semeraro *et al.*, 2017 ▸). Specifically, the model applies a core–shell description of the cell’s body, composed of an ellipsoidal cytoplasmic space and a multilayered cellular wall, and includes contributions from flagella in terms of self-avoiding polymer chains (Doi & Edwards, 1988 ▸). The last component was found to significantly contribute at intermediate to high magnitudes of the scattering vector **q**.

Continuing our efforts to use elastic scattering techniques for exploiting the ultrastructure of *E. coli* led us to perform SAXS experiments on *E. coli* ATCC 25922 with either regular or short flagella, and the ATCC flagellum-free mutant Δ*fliC* with the surprising result of basically superimposable scattering patterns (see Fig. S1 in the supporting information). This prompted us to thoroughly revise our analytical form factor model on the basis of robust estimates of the molecular composition of the bacteria and their structural integration. These estimates include the sizes, volumes, concentrations and distributions of all major bacterial components.

Briefly, the most important changes of our revised bacterial model are as follows: (i) constraints for the average scattering length densities (SLDs) of different cell compartments were derived by considering their constituting macromolecules as separate bodies, including estimates of SLDs of the metabolome and membrane; (ii) contributions from flagella were replaced by the oligosaccharide cores of lipopolysaccharide (LPS), modeled as grafted polymers; and (iii) variations of the inter-membrane distance are modeled by a log-normal probability distribution function (PDF) along with removing the negligible polydispersity over the cell radius from the model.

The model was tested against USAXS/SAXS and very small angle neutron scattering (VSANS)/SANS data at ten different contrasts of *E. coli* ATCC 25922, yielding highly satisfactory fits over the complete range of recorded scattering vector magnitudes (3 × 10^−3^ < *q* < 7 nm^−1^). These tests also include a more complex model, considering a heterogeneously structured cytosol and including specifically the scattering originating from ribosomes. Our analysis showed, however, that the ribosome contribution to the overall scattering is overwhelmed by that of the cell wall. The new model was also successfully applied to USAXS/SAXS data of *E. coli* K12, previously used for devising our multi-scale model (Semeraro *et al.*, 2017 ▸), as well as the fimbra-free JW4283 and the strain Nissle 1917, revealing distinct differences in ultrastructural features.

The paper is structured as follows. First we briefly summarize the experimental methods and samples, before we detail the revised modeling in Section 3, including a comprehensive list of compositional data in the supporting information. Section 4 describes an analytical model for scattering from a heterogeneous cytosol accounting for ribosomes and Section 5 summarizes the involved parameters and applied optimization strategy. Results of applying the modeling to experimental data of five different *E. coli* strains are described and discussed in Section 6, before we conclude in Section 7.

## 2. Materials and methods   

### 2.1. Bacterial samples   

Bacterial colonies of *E. coli* strains ATCC 25922, K12 5K, K12 JW4283 (Baba *et al.*, 2006 ▸) and Nissle 1917 (Sonnenborn, 2016 ▸) were grown in lysogeny broth (LB)–agar (Carl Roth, Karlsruhe, Germany) plates at 310 K. Overnight cultures (ONCs) were derived from these colonies by inoculating a single colony in 3 ml of LB medium (Luria/Miller, Carl Roth) in sterile polypropylene conical tubes (15 ml), allowing for growth under aerobic conditions for 12–16 h in a shaking incubator at 310 K. Main cultures were prepared by suspending an aliquot of the ONCs in 10 ml of LB medium in 50 ml sterile polypropylene conical tubes, allowing for bacterial growth under the same conditions as applied to ONCs up to the middle of the exponential growth phase. Cells were then immediately washed twice and re-suspended in nutrient-free and isotonic phosphate-buffered saline (PBS) solution (phosphate buffer 20 m*M*, NaCl 130 m*M*) at pH 7.4 (Sigma Aldrich, Vienna, Austria). Turbidity measurements were used to control the bacterial concentration. Optical density values at wavelength λ = 600 nm (OD_600_) were acquired with the spectrophotometer Thermo Spectronic Genesys 20 (Thermo Fisher Scientific, Waltham, MA, USA) [OD_600_ = 1 ≃ 8 × 10^8^ colony forming units (CFU) per millilitre]. In these samples, 1 CFU corresponds to one single cell. In the case of samples containing D_2_O, bacterial suspensions were washed twice with either PBS or 90 wt% D_2_O PBS solutions, in order to obtain two concentrated stock solutions for both buffer conditions. These two stocks were mixed and diluted down to the required bacterial concentrations and D_2_O contents according to the experimental settings.

The preparation of the ATCC samples was conducted with the maximum care in order to preserve the integrity of the flagella. ATCC cells with mechanically fragmented flagella were prepared by shearing the suspension five times through a 3 ml syringe equipped with a 22-gauge needle, as described by Turner *et al.* (2012 ▸). The suspension was washed in PBS to eliminate flagellum fragments in the supernatant.

The ATCC 25922 Δ*fliC* strain was constructed by phage transduction as described by Silhavy *et al.* (1984 ▸). The P1vir phage was propagated on strain YK4516 (Komeda *et al.*, 1980 ▸) using the plate lysate method. YK4516 contains a Tn10 insertion in *fliC* (fliC5303::Tn10) and was purchased from The Coli Genetic Stock Center (Yale University, New Haven, CT, USA). Strain ATCC 25922 served as recipient. Transductands were selected on tetracycline-containing plates (10 µg ml^−1^) and the *fli^−^* phenotype was confirmed by spotting on semi-solid agar plates (0.3% agarose).

### 2.2. Experimental setup   

#### 2.2.1. USAXS   

USAXS/SAXS measurements were performed on the TRUSAXS beamline (ID02) at the ESRF, Grenoble, France. The instrument uses a monochromatic beam that is collimated in a pinhole configuration. Measurements were performed with a wavelength of 0.0995 nm and sample-to-detector distances of 30.8, 3.0 and 1.2 m, covering a *q* range of 0.001–7 nm^−1^ (Narayanan *et al.*, 2018 ▸). The measured two-dimensional scattering patterns were acquired on a Rayonix MX170 detector, normalized to absolute scale and azimuthally averaged to obtain the corresponding one-dimensional USAXS/SAXS profiles. The normalized cumulative background from the buffer, sample cell and instrument was subtracted to obtain the final *I*(*q*). Samples with a bacterial concentration of ∼10^10^ cells ml^−1^ were measured at 310 K and contained in quartz capillaries of 2 mm diameter, mounted on a flow-through setup in order to maximize the precision of the background subtraction.

#### 2.2.2. VSANS   

VSANS/SANS measurements were acquired on the D11 instrument at the Institut Laue–Langevin (ILL), Grenoble, France, with a multiwire ^3^He detector of 256 × 256 pixels (3.75 × 3.75 mm). Four different setups (sample-to-detector distances of 2, 8, 20.5 and 39 m with corresponding collimations of 5.5, 8, 20.5 and 40.5 m, respectively), at a wavelength λ = 0.56 nm (Δλ/λ = 9%), covered a *q* range of 0.014–3 nm^−1^. To reach very low *q*, a combination of large wavelength (λ = 2.1 nm), two focusing MgF_2_ lenses and a mirror to cancel deleterious gravity effects (loss of neutrons in the collimation and loss of resolution due to gravity smearing on the detector) were used; the setup is described by Cubitt *et al.* (2011 ▸). Samples (concentration ∼10^10^ cells ml^−1^) were measured at 310 K and contained in quartz Hellma 120-QS banjo-shaped cuvettes of 2 mm pathway. They were mounted on a rotating sample holder, which prevented the bacteria from sedimenting. Data were reduced with the *Lamp* program from ILL, performing flat-field, solid angle, dead time and transmission correction, normalizing by incident flux (via a monitor), and subtracting the contribution from an empty cell. The experimental setup and data are available at https://doi.org/10.5291/ILL-DATA.8-03-910.

#### 2.2.3. In-house SAXS   

A SAXSpace compact camera (Anton Paar, Graz, Austria) equipped with an Eiger R 1 M detector system (Dectris, Baden-Daettwil, Switzerland) was used for laboratory SAXS experiments. Cu *K*α (λ = 1.54 Å) X-rays were provided by a 30 W-Genix 3D microfocus X-ray generator (Xenocs, Sassenage, France). Samples were taken up in glass capillaries (diameter: 1 mm; Anton Paar) and equilibrated at 310 K for 10 min prior to measurement using a Peltier-controlled sample stage (TC 150, Anton Paar). The total exposure time was 30 min (6 frames of 5 min), setting the sample-to-detector distance to 308 mm. Data reduction, including sectorial data integration and corrections for sample transmission and background scattering, was performed using the program *SAXSanalysis* (Anton Paar).

#### 2.2.4. Dynamic light scattering   

Measurements were performed using a Zetasizer Nano ZSP (Malvern Panalytical, Malvern, UK). ATCC 25922 and K12 5K samples (concentration ∼10^7^ cells ml^−1^) were suspended in glass cuvettes of 1 cm path length and equilibrated at 310 K. This bacterial concentration provided an optimal signal-to-noise ratio and avoided multiple-scattering effects. Data were automatically analyzed by the supplied software (Malvern), which, via a standard cumulant analysis, provided a monomodal probability distribution of the bacterial population as a function of the hydrodynamic radius *R*_H_. Each distribution had an average polydispersity index of ∼0.25, due to the variations of the cell lengths during growth and division. Average *R*_H_ values and the associated errors were calculated from 18 measurements (six frames of three different sample volumes). The absence of energy sources in PBS and vigorous vortexing of the samples (fragmentation of flagella) minimized cell motility, making Brownian random walk the dominant dynamics of this sample.

## 3. Overall scattering contributions and compositional modeling   

**Figure 1:**
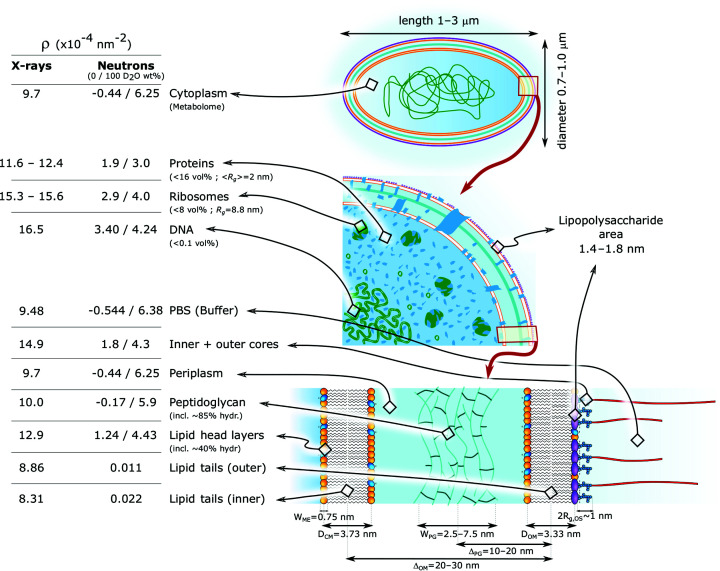
Schematic of *E. coli* structure and composition, including typical sizes, as well as X-ray and neutron SLDs of the most relevant constituents. The bacterial shape is conveniently modeled by an ellipsoid, as detailed by Semeraro *et al.* (2017 ▸).

A holistic description of elastic scattering from complex Gram-negative prokaryotic cells can be derived by considering first their prevalent molecular and supramolecular components, each of them having a well defined range of lengths, volumes and densities, which serve as a guide to construct a comprehensive scattering-form-factor model. The here-applied scattering contribution estimates are based on the latest experimental and computational reports on *E. coli*, including isolated cell components. Specifically, we used compositional and structural information about *E. coli* and its cell wall (Neidhardt *et al.*, 1990 ▸; Seltmann & Holst, 2002 ▸; Schwarz-Linek *et al.*, 2016 ▸); the cytoplasmic space and its components (Zimmerman & Trach, 1991 ▸; Tweeddale *et al.*, 1998 ▸; Prasad Maharjan & Ferenci, 2003 ▸; Bennett *et al.*, 2009 ▸; Guo *et al.*, 2012 ▸; Lebedev *et al.*, 2015 ▸); the bacterial ultrastructure (Hobot *et al.*, 1984 ▸; Beveridge, 1999 ▸; Matias *et al.*, 2003 ▸); the lipid membrane composition and structure (De Siervo, 1969 ▸; Oursel *et al.*, 2007 ▸; Lohner *et al.*, 2008 ▸; Pandit & Klauda, 2012 ▸; Kučerka *et al.*, 2012 ▸, 2015 ▸; Leber *et al.*, 2018 ▸); the LPS specifics (Heinrichs *et al.*, 1998 ▸; Müller-Loennies *et al.*, 2003 ▸; Kučerka *et al.*, 2008 ▸; Kim *et al.*, 2016 ▸; Rodriguez-Loureiro *et al.*, 2018 ▸; Micciulla *et al.*, 2019 ▸); the periplasmic space (Burge *et al.*, 1977 ▸; Labischinski *et al.*, 1991 ▸; Pink *et al.*, 2000 ▸; Gan *et al.*, 2008 ▸); and the external bacterial components (Yamashita *et al.*, 1998 ▸; Whitfield & Roberts, 1999 ▸; Stukalov *et al.*, 2008 ▸; Turner *et al.*, 2012 ▸). All this information has been condensed into SLDs ρ, which are summarized in Fig. 1 ▸. For a detailed description, see the supporting information (SI).

**Figure 2:**
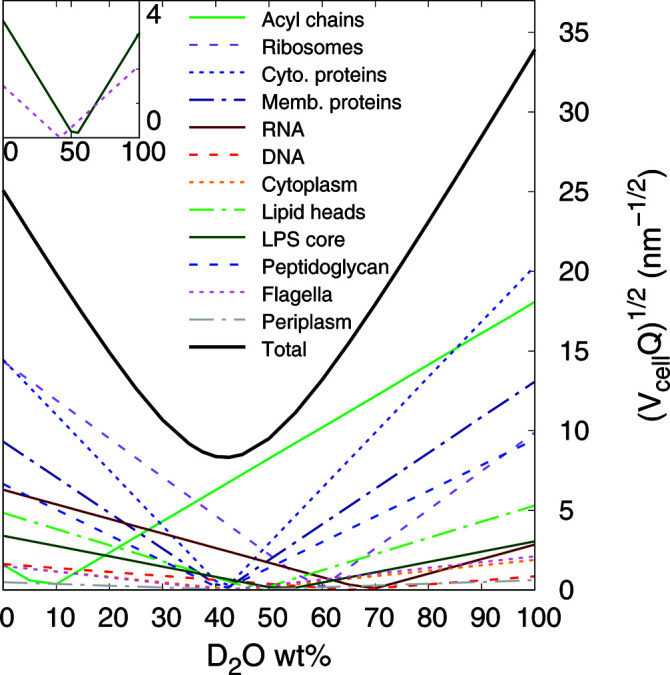
Square root of the estimated Porod invariant *Q* as a function of D_2_O wt%, calculated for each component using equation (1) and multiplied by the cell volume. The inset shows the differences between the contribution of the LPS oligosaccharide cores (solid green) and flagella (dashed pink).

The benefit of this detailed description becomes clear when considering the ability of SANS to nullify or enhance contrast for a given molecular entity, depending on the applied H_2_O/D_2_O ratio. For homogeneous scatterers in a dilute regime, *i.e.* when their volume fraction 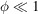, the forward scattering intensity, *I*(0), is related to the scattering invariant, *Q*, as
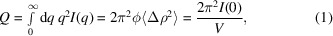
where *V* is the volume of the scatterer and 〈Δρ^2^〉 is the average squared scattering contrast (Porod, 1982 ▸). For inhomogeneous systems, such as complex live cells, the average contrast can be calculated as a volume-fraction-weighted average of the contrast of each cell compartment/species 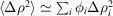, where ϕ_*i*_ is the volume fraction of the *i*th component. Hence, the estimates ϕ_*i*_ and Δρ_*i*_ enable us to approximate *Q* for all bacterial components [Fig. 2 ▸; see also Nickels *et al.* (2017 ▸)]. This approximation leads to a ‘matching’ point of the entire cell at about 40 wt% D_2_O. At higher D_2_O content the total scattering intensity is increasingly dominated by the acyl chains of the membrane lipids, because they are devoid of water. Toward lower D_2_O contents, cytoplasmic components, such as ribosomes and proteins, are the dominating scattering contributors in turn.

### 3.1. Multi-scale scattering model   

As reported previously (Semeraro *et al.*, 2017 ▸), the main body of *E. coli* can be described in terms of the scattering amplitude of an ellipsoid with multiple shells (multi-core–shell): 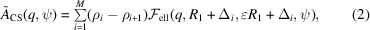
where ρ_*i*_ are the SLDs given by the compositional *E. coli* averages of each shell of width Δ_*i*_ (ρ_*M*+1_ is the SLD of the buffer); ψ is the angle related to every possible orientation of a prolate in suspension; *R*_1_ is the minor radius of the cytoplasmic core (CP) and ɛ > 1 is the ratio between the major, ɛ*R*_1_, and minor radii of the ellipsoid. Furthermore, 
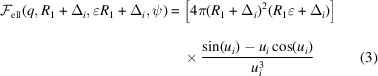
is the product of the volume and normalized scattering amplitude of the ellipsoid (Pedersen, 1997 ▸), where 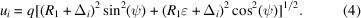
Despite the fact that cylinders would be more realistic bacterial shape models, prolates do not incur instabilities during fitting and differences between a prolate or cylinder geometries are negligible in reciprocal space (Semeraro *et al.*, 2017 ▸). A significant difference from the previous model (Semeraro *et al.*, 2017 ▸) relates to the ρ_*i*_ values used as fitting constraints. Here, we considered, given the available *q* range, scattering contributions from every macromolecular species (proteins, ribosomes, DNA *etc*.) individually for calculating the average SLDs. This affects, compared with our previously used values, in essence the ρ_*i*_ estimates of the cytoplasm – now based on metabolomic analysis – and the phospholipid membranes (Fig. 1 ▸). In contrast to elastic scattering experiments on lipid-only mimics, structural parameters of each single bilayer cannot be resolved in the context of a whole-cell analysis (Semeraro *et al.*, 2020 ▸). Hence, the Δ_*i*_ and ρ_*i*_ values of both membranes were considered as fixed parameters (see Fig. 1 ▸; more details about Δ_*i*_ and ρ_*i*_ are given in the SI).

The volume fraction of the bacterial suspensions was ≤0.007; hence the presence of an inter-cellular structure factor of interactions is unlikely.

Arguably the most distinct differences compared with the former model result from flagella, whose scattering was previously added in terms of a polymer-like structure factor yielding significant contributions to *I*(*q*) for *q* > 0.06 nm^−1^ (Semeraro *et al.*, 2017 ▸). Surprisingly, however, a comparison of SAXS data of native ATCC 25922, ATCC with physically broken, short flagella (Turner *et al.*, 2012 ▸) and the flagellum-free Δ*fliC* ATCC mutant showed indistinguishable scattering intensities in the *q* range previously thought to be dominated by flagella (Fig. S1). Consequently, flagella do not contribute significantly to the *E. coli* scattering signal. Note that the integrity of flagellar filaments in common bacterial cultures is not guaranteed, as excessive centrifuging or careless sample manipulation steps easily lead to their fragmentation (Schwarz-Linek *et al.*, 2016 ▸). Even if we cannot guarantee that the reference ATCC sample possessed fully intact flagella, the bacterial suspensions used in this work were prepared with the utmost care. The same sample preparation protocol allowed us to obtain motile bacteria (Semeraro *et al.*, 2018 ▸), suggesting that the flagellar integrity was preserved at least to some degree.

Attempting to rectify the missing scattering intensities in our multi-scale model led us to consider contributions originating from the oligosaccharide (OS) inner and outer cores. Initially, the function 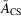 was modified by a new shell describing the OS cores, which resulted in nonphysical results (Fig. S2). In particular, ρ_X-ray_ ≃ 15 × 10^−4^ nm^−2^ for the OS layer suggested that water is expelled, which is inconsistent with neutron reflectometry experiments on supported LPS layers showing that the hydration of the inner and outer cores ranges from 40 to 80 vol% (Clifton *et al.*, 2013 ▸; Rodriguez-Loureiro *et al.*, 2018 ▸; Micciulla *et al.*, 2019 ▸). We therefore decided to model the OS cores in terms of grafted blocks on the outer cell surface. Each core was approximated by a Gaussian chain polymer, entailing the application of the polymer-grafted colloid formalism (Pedersen & Gerstenberg, 1996 ▸). The scattering form factor of such a system is given by (Pedersen, 2000 ▸)
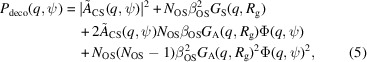
where *N*_OS_ is the number of OS cores and β_OS_ = *V*_OS_(ρ_OS_ − ρ_BF_) is the product of each volume and SLD contrast to the buffer,
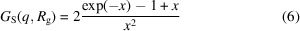
is the structure factor of a Gaussian chain, and 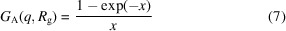
its scattering amplitude. *x* = (*qR*_g_)^2^, where *R*_g_ is the radius of gyration of the OS core. The term Φ(*q*, ψ) in equation (5) is related to the ‘cross-term’ resulting from a uniform distribution of OS cores all along the ellipsoidal surface of a single bacterium (Pedersen, 2000 ▸) and is given by
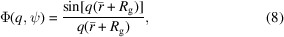
where, in accordance with (4), 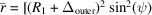 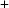 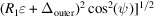. Here, *R*_outer_ = *R*_1_ + Δ_outer_ is the radius describing the external surface of the cell.

The total scattering intensity for a suspension of live *E. coli* cells of number density *n* then reads as
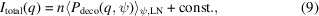
where 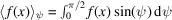 is the orientational average and 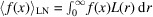 describes the polydispersity of the thickness of the periplasmic space. Specifically, we applied a log-normal distribution function *L*(*r*). The constant in equation (9) takes into account scattering background at high *q* originating from unidentified contributions. The log-normal distribution of the periplasmic thickness takes care of the lower cut-off in intermembrane distance fluctuations, given by the finite size of the cell-wall architecture. Note that cell size variations were not considered due to overparameterization; in fact, the polydispersity over the cell radius brought about insignificant changes in the middle to high *q* range, *i.e.* *q* ≥ 0.05 nm^−1^.

## 4. Considering a heterogeneously structured cytosol   

It is legitimate to question whether the internal cytosolic structure can be resolved at least in part by SAXS/SANS. In order to address this issue, we derived a more complex analytical scattering function that can be tested against experimental data.

Let us start by considering the general case of a sphere (radius: *R*_0_; SLD: ρ_0_) suspended in a medium of ρ_M_, containing *N* smaller identical spherical beads (*R*_1_, ρ_1_), where the relative distance between two beads *r*_*i*_ < *R*_0_ − *R*_1_. The scattering amplitude of this system is
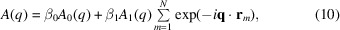
where β_0_ = (ρ_0_ − ρ_M_)*V*_0_ and β_1_ = (ρ_1_ − ρ_0_)*V*_1_, and *V*_0_, *V*_1_, *A*_0_ and *A*_1_ are, respectively, the volumes of the sphere and a single bead, and the normalized scattering amplitudes of the sphere and a bead. The vector **r**_*m*_ defines all relative distances between the internal beads. The total form factor [*P*(*q*) = |*A*(*q*)|^2^] then reads as
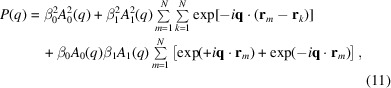
where the first term is the form factor of the sphere; the second term is the total scattering intensity of the *N* internal beads; and the third describes the cross-term between the sphere and the beads. An analogous approach was reported for modeling the internal structure of a ‘biphasic’ copolymer system (Keerl *et al.*, 2009 ▸). Here, by assuming 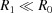 and *N* → ∞, one can approximately describe the interior of this sphere as a macroscopic canonical system. This enables the application of the canonical ensemble average 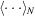 to the summations of equation (11) (Klein & D’Aguanno, 1996 ▸), leading to
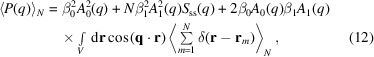
where *S*_ss_(*q*) corresponds to the structure factor of interactions among the beads. The summation within the integral defines the microscopic density of *N* beads. Its ensemble average corresponds to the single-particle density, which, because of the translational invariance of a homogeneous system, is equal to the average bead density *N*/*V*_0_ (Klein & D’Aguanno, 1996 ▸). Hence, the whole integral is simply equal to *NA*_0_(*q*), yielding the final form of the spherical bead system form factor: 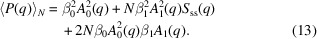



The cross-term is thus modulated by the normalized scattering amplitude of the sphere, *A*_0_(*q*), which is equivalent to a convolution of a homogeneous distribution of beads within the volume of a sphere of radius *R*_0_. Importantly, this final form of the cross-term does not depend on the original volume of the sphere hosting the small spheres. That is, even if the beads are constrained within a specific region of the sphere – such as in the case of ribosomes, which mainly partition into the non-nucleoid region of the cytoplasm – the scaling of this cross-term does not change.

In the next step, we translate the spherical bead system to the case of a heterogeneously structured bacterial cytosol. This requires a few approximations. First of all, encouraged by the fact that the scattering intensity scales proportionally to the square of the particle volume, we focus on contributions originating from ribosomes. More specifically, ribosomes with a volume *V*_rb_ ≃ 2800 nm^3^, a total number *N*_rb_ ≃ (1 − 6) × 10^4^ and *R*_g_ = 8.77 nm (Zimmerman & Trach, 1991 ▸; Lebedev *et al.*, 2015 ▸) should be the dominating cytosolic scatterers in the case of X-rays or neutrons in the absence of heavy water (Fig. 2 ▸ and SI, for cytoplasmic composition and volume fractions). Secondly, we approximate the scattering of ribosomes at very low *q*, *i.e.* in the Guinier regime up to the first minimum of the scattering form factor, by the scattering of an ‘effective’ sphere of *V*_rb_ = 2800  nm^3^ with *R*_rb_ = 11 nm. Thirdly, we assume that mixed interactions with macromolecules of different size, shape and net charge (cytosolic proteins) lead to an overall effective *S*_ss_(*q*) ≃ 1. Furthermore, we simplify the cross-term modulation by a compact ellipsoid, neglecting that ribosomes sequester to the non-nucleotide region, and do not include the grafted OS cores in the cross-term.

Then the final form of the scattered intensity, considering contributions from ribosomes, is given by
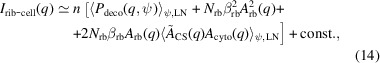
where β_rb_ = *V*_rb_(ρ_rb_ − ρ_cyto_), *A*_rb_ is the normalized scattering amplitude of ribosomes approximated by an equivalent sphere and *A*_cyto_ is the normalized scattering amplitude of a prolate describing the cytoplasmic space (*i.e.* only the central part of the core–shell system defining 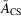).

## 5. Parameterization and optimization strategy   

All parameters needed to describe elastic SAS from *E. coli* are summarized in Table 1 ▸. In general we differentiate between parameters that either do or do not depend on the individual experiment (*e.g.* sample concentration, scattering contrast). Experiment-specific parameters are termed ‘local’, while others are designated as ‘global’ parameters.

Parameters describing structural details of the cytoplasmic membrane (CM) and outer membrane (OM) were fixed according to values reported from experiments and simulations on membrane mimic systems of *E. coli* cell membranes (De Siervo, 1969 ▸; Oursel *et al.*, 2007 ▸; Lohner *et al.*, 2008 ▸; Pandit & Klauda, 2012 ▸; Kučerka *et al.*, 2012 ▸, 2015 ▸; Leber *et al.*, 2018 ▸), as detailed in Table 2 ▸ (see also Fig. 1 ▸ and the SI). This decision can be rationalized by the lack of distinct scattering features for *q* > 0.27 nm^−1^, corresponding to distances of ∼20 nm. Consequently, structural features ≤20 nm are, although contributing to the overall scattering, difficult to resolve with appropriate accuracy. Note that membrane proteins are treated similarly to proteins in other compartments as bodies adding individually to the scattered intensity and thus do not contribute to the average SLDs of the inner and outer membranes. Compared with the overall scattering arising from the cell body, their overall contribution can be shown to be negligible. Similarly, the width of the peptidoglycan layer (PG), *W*_PG_, and the radius of gyration of the OS core, *R*_g,OS_, were fixed to the values reported in Table 2 ▸ after analyzing their contributions. *W*_PG_ values of ∼6 nm have been reported (Matias *et al.*, 2003 ▸), and simulations in the range 2.5–7.5 nm (Labischinski *et al.*, 1991 ▸) led to insignificant variations of ρ_PG_. Furthermore, our estimates show that *R*_g,OS_ < 1 nm (see SI). However, variations of its value within this constraint do not lead to significant changes within our scattering model, because *R*_outer_ ≫ *R*_g,OS_.

Finally, the ratio between major and minor ellipsoidal radii, ɛ, was fixed for the ATCC and K12 strains. This choice was driven by the lowest available *q* range, which does not reach the Guinier plateau of the bacterial scattering and thus does not allow for an accurate determination of the cell length. We therefore used dynamic light scattering to first estimate the average length, *L*_c_, from 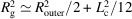, using *R*_g_ ≃ *R*_H_ and typical values reported for *R*_outer_ in the literature. This led to 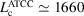
 nm and 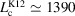
 nm. The resulting ɛ = *L*_c_/(2*R*_outer_) values were subsequently refined in some test runs of the optimization procedure for USAXS/SAXS data with *R*_outer_ as adjustable parameter and then fixed for the detailed USAXS/SAXS and VSANS/SANS analysis, yielding the values reported in Table 2 ▸.

Owing to the complexity of the system and the high number of parameters, optimization of the adjustable parameters was performed with a Monte Carlo genetic selection algorithm (Banzhaf *et al.*, 1998 ▸). In brief, the algorithm is schematized in a series of steps that are repeated at every cycle (*generation*). As step zero, nine low-discrepancy sequences (quasi-random numbers) were created for each parameter (*gene*) within specific boundaries, based on compositional estimates (see the SI). Nine sets of parameters (*individuals*) were then used as input for testing an equal number of possible scattering intensity curves (step 1, *evaluation*). This involved the calculation of nine standard weighted chi-squared values as
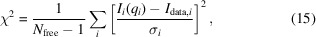
where *N*_free_ is the number of free parameters and σ_*i*_ is the error associated with the measured *I*_data,*i*_ at a given *q*_*i*_. Only the four *individuals* with the lowest χ^2^ values were then selected (step 2, *selection*) to generate the first *offspring*, consisting of eight new *individuals* that were created by randomly shuffling the *genes* of the four selected parents (step 3, *recombination*). The new ninth *individual* was a copy of the parent with the lowest χ^2^ value (rule 1: *inheritance of the best*). After the *recombination*, each *gene* had a finite probability of being altered (step 4, *mutation*). In addition, there was a finite probability of replacing an entire *individual* with a brand new set of randomly created *genes* (rule 2: *the stranger*). This construction of the new *offspring* ended the first cycle, and each *individual* was again evaluated on the basis of the χ^2^ values (jumping back to step 1). The process was repeated until the changes of the lowest 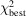 were less than 0.5% for 25 consecutive *generations*.

The *mutation* step and rule 2 allow the algorithm to skip possible local minima in the χ^2^ landscape, whereas rule 1 enables a fast convergence of the fitting. Note that, except for the creation of the initial set of *individuals*, pseudo-random numbers were used in the whole algorithm for the decision-making processes of the *recombination* and *mutation* steps, and for rule 2. Note also that a larger initial population (>9 *individuals*) did not result in a gain of computational time. Each new pseudo-random *gene* was always constrained within the initial boundaries, in order to ensure the preservation of a physical meaning of the results. In total, each scattering curve was fitted 500 times, and only converging fittings (convergence criterion 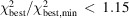) were used to retrieve average values and standard deviations for each parameter.

In the case of SANS, the *q*-dependent instrumental smearing was additionally taken into account. This was accomplished with a standard convolution 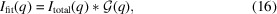
where 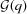 is a normalized Gaussian profile of width Δ*q*(*q*). The Δ*q*(*q*) values as a function of *q* were fixed parameters during the fitting and were provided by the D11 primary data treatment. In contrast, the effect of the instrumental smearing was negligible for SAXS data.

## 6. Results and discussion   

### 6.1. SAXS/SANS global analysis   

**Figure 3:**
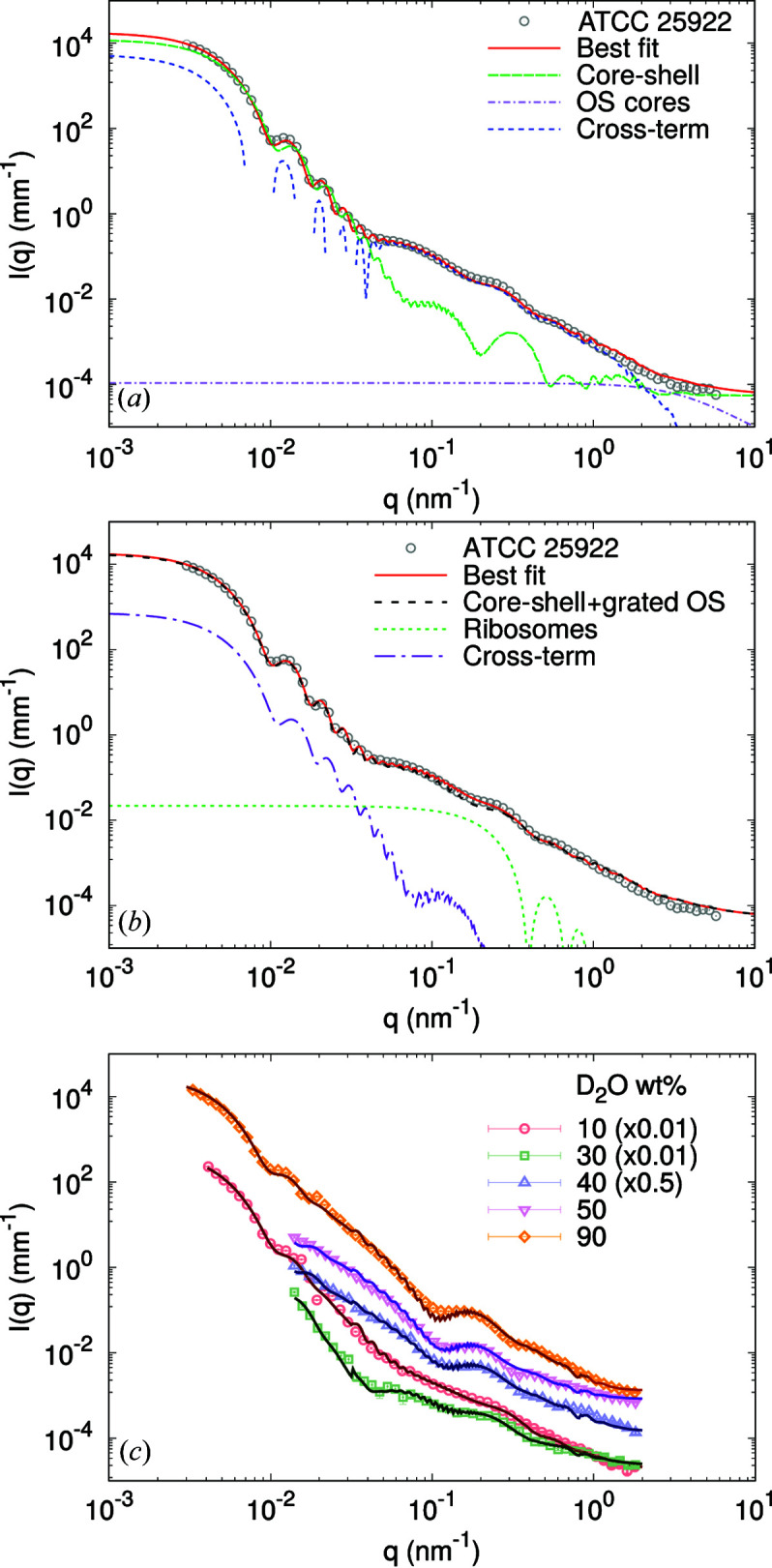
(*a*) USAXS/SAXS data analysis of *E. coli* ATCC 25922 using equation (9), highlighting contributions from different terms (negative values of the cross-term are not shown). (*b*) Alternative analysis of the same data using equation (14), showing contributions from ribosomes. Comparison with a fit using equation (9) (black dashed line) shows negligible differences. (*c*) VSANS/SANS data of the same strain at selected D_2_O contrasts (see Fig. S4 for additional neutron data). Scattering curves were scaled for better visibility.

The revised multi-scale model was tested against USAXS/SAXS and VSANS/SANS data on *E. coli* strain ATCC 25922. Ten SANS data sets with different contrast conditions were collected, varying D_2_O from 0 to 90 wt% (increments of 10 wt%). Results of the combined SAXS/SANS data analysis are reported in Fig. 3 ▸ and Tables 3 ▸ and S1. Fig. 3 ▸(*a*) highlights the different contributions from the multi-core–shell model, the OS cores and the sum of the two cross-terms [see equation (5)] in the USAXS and SAXS regimes. The scattering contribution from the core–shell function, *i.e.* cell body plus cell wall, dominates over the scattering intensity originating from the OS cores, owing to the huge difference in mass. However, the cross-term, being a function of the whole cell surface, is mainly responsible for modulating the scattered intensities between *q* ≃ 0.1 nm^−1^ and *q* ≃ 0.3 nm^−1^. This leads to an average slope between *q* ^−1.5^ and *q* ^−2^ in this regime, which is a typical signature of grafted systems, also called ‘blob scattering’ (Pedersen, 2000 ▸). Previously, this regime was taken to be dominated by flagella, described by a self-avoiding-walk polymer term (Semeraro *et al.*, 2017 ▸). This, however, does not describe the apparent change of slope at *q* ≃ 0.04 nm^−1^ and the scattering feature at *q* ≃ 0.1 nm^−1^, which appears to be specific to the ATCC strain (see Fig. S3) but not K12 (see below). Hence, the OS-core cross-term enables a full description of the *q* range between 0.03 and 0.2 nm^−1^.

Attempts to fit the same data with the more complex model accounting for ribosomes [equation (14)] demonstrated insignificant scattering contributions from the macromolecules [Fig. 3 ▸(*b*)]. In particular, the goodness of fit and results for the common adjustable were identical within the error of the analysis. Thus, the ribosome term, along with its related cross-term, is negligible compared with the cell-wall contribution. Note that only SAXS or SANS data at the lowest wt% of D_2_O are sensitive to test for this contribution. SANS data are dominated by the acyl-chain contribution at higher heavy-water content (Fig. 2 ▸). Furthermore, ribosomes are made up of amino acids and RNA, which will be differently matched, thus challenging the analysis. Note that *N*_rb_ was the only adjustable parameter for this analysis. *V*_rb_ and *R*_rb_ values were fixed as detailed in Section 4. Interestingly, the outcome of this test resulted in a much smaller number of ribosomes (*N*_rb_ ≃ 500) than our estimate of *N*_rb_ ≃ 10^4^ following Zimmerman & Trach (1991 ▸) (see the SI). This is possibly related to the low contrast of these molecules in the local cytoplasmic environment. As the scaling is proportional to *N*_rb_(ρ_rb_ − ρ_CP_)^2^, small differences in the effective contrast easily skew the determination of the number of macromolecules.

Importantly, this analysis demonstrates not only that the effective scattering signal from ribosomes is negligible but also that similar considerations can be applied to the other cytoplamic components. However, because of their smaller size (proteins) or smaller volume fraction (DNA and RNA), they will contribute even less to the overall scattered intensity. Hence, elastic scattering techniques are not suitable for discriminating differently structured compartments within the cytosol in live bacterial cells. The same applies to membrane proteins (see above) or proteins present in the periplasmic space and peptidoglycan layer.

**Figure 4:**
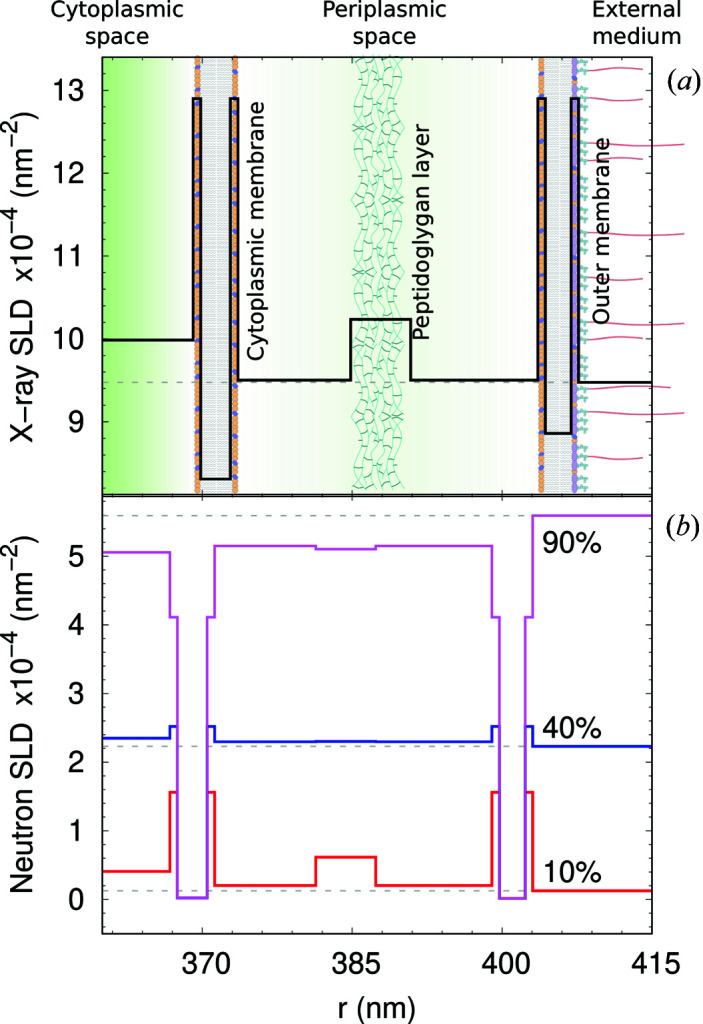
(*a*) X-ray SLD profile of the bacterial ultrastructure of ATCC 25922 strain, corresponding to the fit shown in Fig. 3 ▸(*a*). The panel highlights the average positions of both cytoplasmic and outer membrane, and the peptidoglycan layer. The abscissa describes the distance from the cell center along the minor radius *R*. (*b*) Selected neutron SLD profiles of the same strain [*cf*. Fig. 3 ▸(*c*)]. See also Table S1.

Analysis of selected VSANS/SANS data at selected contrasts is presented in Fig. 3 ▸(*c*) (the entire set of neutron scattering data and fits is presented in Fig. S4). Clearly, fits using equation (9) neatly capture all changes of scattered intensities upon varying D_2_O concentrations, lending strong support to our modeling approach. The resulting parameters forming the X-ray and neutron SLD profiles of the bacterial envelope are summarized in Fig. 4 ▸, again at selected neutron contrasts (see Fig. S5 for all neutron SLD profiles). Small differences between distances in X-ray and neutron profiles, as well as global parameters (Table 3 ▸), are due to biological variability of the samples, but are, with the exception of *N*_OS_, still within the confidence range of the results. At 10 wt% D_2_O, the contrast differences between different slabs are comparable to those obtained from SAXS data. The scattering intensities in these cases were also comparable in terms of scattering features at *q* ≃ 0.1 and 0.3 nm^−1^ (Fig. 3 ▸). In turn, at 40 D_2_O wt% (and similarly up to 90 wt% D_2_O), the contrast of highly hydrated bacterial subcompartments (PG layer *etc*.) is much lower than the major contrast of the hydrophobic regions of the two membranes [Fig. 4 ▸(*b*)]. This characteristic leads to the shift in the scattering feature from *q* ≃ 0.27 nm^−1^ to *q* ≃ 0.2 nm^−1^ [Fig. 3 ▸(*c*)], which is primarily related to the intermembrane distance. This is in good qualitative agreement with the invariant estimation (Fig. 2 ▸), which suggests that the scattering intensity is dominated by the contribution from the acyl-chain region for D_2_O ≥ 40 wt%.

**Figure 5:**
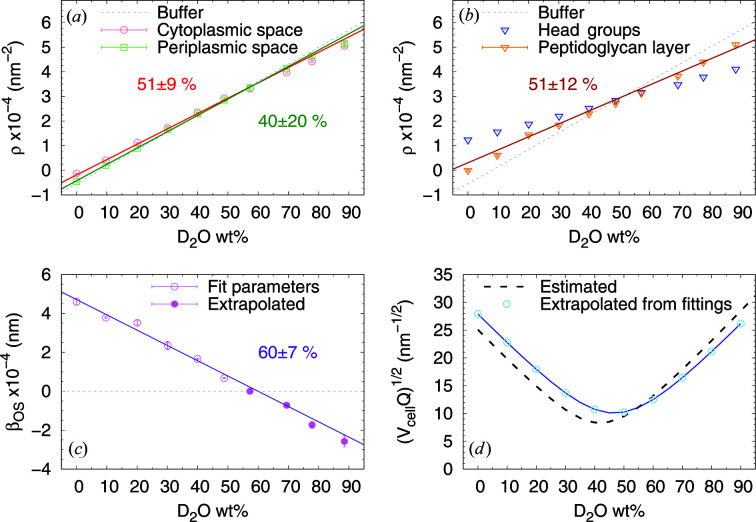
(*a*), (*b*) Plots of the cytoplasm (red circles), periplasm (green squares) and peptidoglycan (orange triangles) SLDs, along with linear fittings and matching points. The SLDs of the phospholipid head-group layers were fixed parameters (blue triangles). (*c*) Plot of the β_OS_ (purple circles) values, along with linear fits and matching points. (*d*) Comparison between estimated scattering invariant and extrapolated forward scattering.

In order to test our modeling strategy, we report the variation of the various SLDs with D_2_O content. Since the solvent freely accesses the cytoplasmic and periplasmic spaces, such plots should display a linear dependency. Indeed, the trends followed the expected behavior, which also enabled us to calculate the match points for the individual compartments [Fig. 5 ▸(*a*)–5 ▸(*c*)]. Note that the SLD values of the hydrated phospholipid headgroups were fixed (Table 2 ▸). In the case of β_OS_, scattering contributions are superseded by the signal originating from the intermembrane distance for D_2_O < 60 wt%. The linear trend for β_OS_ was therefore determined in the range 0−50 wt%, and then extrapolated to higher D_2_O concentrations by using a confidence boundary of 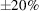. Results from this analysis were used to derive the measured effective invariant as a function of D_2_O wt% [Fig. 5 ▸(*d*)]. The comparison with the estimated *Q* shows a shift in the minimum from the estimated 40 wt% to the measured 50 wt% D_2_O, possibly due to a larger contribution from the components that dominate at D_2_O ≤ 30 wt% (Fig. 2 ▸). On the other hand, these components are the very macromolecules that were proven to have a negligible scattering contribution.

The answer to this paradox can be found in the absence of a net size distinction between macromolecules that scatter individually and are included in SLD averages. Translating the molecular mass distribution from gel filtration of cytosolic proteins (Zimmerman & Trach, 1991 ▸) into a size probability distribution function yields a maximum value at the smallest protein size detected in our experiments (see SI). Hence, it is likely that a fraction of the smallest bacterial proteins need to be included in the parameter ρ_CP_. Our fitted values for ρ_CP_ are in fact larger than the estimated SLDs of the metabolites in the case of both SAXS and SANS analysis (compare Fig. 1 ▸ and Table S1). In any case, the estimated invarant was used only as a valuable guide for our modeling. Obviously, equation (1) loses its validity in the case of dense and crowded suspensions, and a new formula accounting for the each volume fraction should be used for improved estimates (Porod, 1982 ▸).

### 6.2. Comparison between ATCC and K12-related strains   

**Figure 6:**
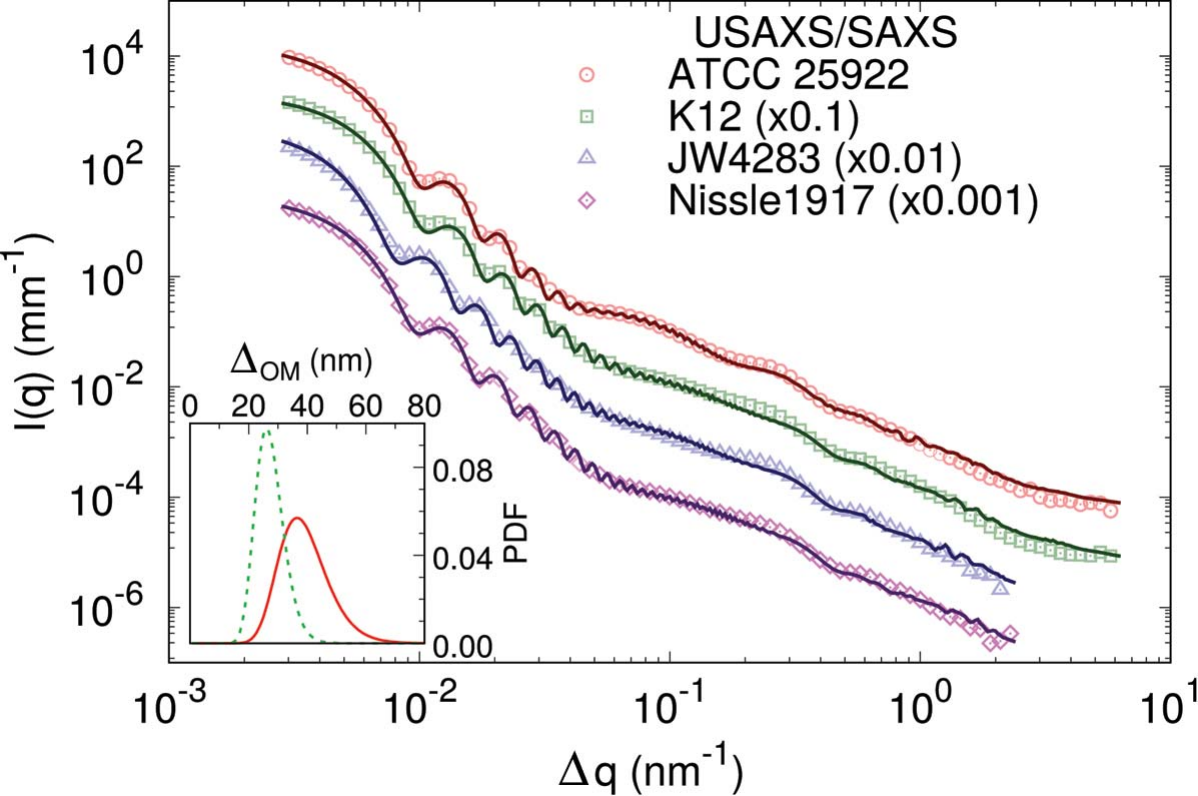
Multi-scale analysis of USAXS/SAXS data of the ATCC, K12, fimbria-free K12 JW4283 and Nissle 1917 strains. The inset shows the plots of the log-normal PDF of Δ_OM_ values for ATCC (solid red) and K12 (dashed green) strains. The PDFs of JW4283 and Nissle 1917 are comparable to K12 and are thus not shown.

After successful verification of our revised multi-scale model for live *E. coli* ATCC, we tested whether the new model is also applicable to other *E. coli* strains. Fig. 6 ▸ shows the USAXS/SAXS data of the K12 5K, JW4283 and Nissle 1917 strains in comparison to ATCC. Strikingly, the scattering patterns of K12 5K, JW4283 (which is fimbria free) and Nissle 1917 are superimposable for *q* > 0.06 nm^−1^, suggesting that the main ultrastructural features are conserved in these strains and confirming that the presence of fimbriae does not contribute to SAXS. Note also that our previous model would perfectly fit all K12 strains. Different minimum positions of the scattered intensities at lower *q* values are due to the different sizes of the different strains, instead.

Importantly, our new model is capable of fitting all strains, as demonstrated by the overall excellent agreement with experimental data (Fig. 6 ▸). Structural parameters resulting from this analysis are reported in Tables 4 ▸ and S2. Most of these parameters are of comparable magnitude. Significant differences concern the cell size (*R*, ɛ) – as observed in the different positions of the scattering minima (Fig. 6 ▸), the number of OS cores (*N*_OS_) and the intermembrane distance (Δ_OM_, σ_OM_). The last is related to the actual periplasmic space thickness via Δ_OM_ = (2*W*_ME_ + *D*_CM_ + *D*_OM_)/2 (see Fig. S6 for the X-ray SLD profiles). Both periplasmic thickness and its fluctuation are smaller for K12-related strains than for ATCC. Note that Δ_OM_ for K12 5K is consistent with our previously reported value for a similar strain (Semeraro *et al.*, 2017 ▸). Despite the different Δ_OM_ and σ_OM_ for ATCC and K12 strains, the magnitude of the relative fluctuations σ_OM_/Δ_OM_ ≃ (0.16– 0.22) is roughly conserved.

Considering cell size differences we find, according to the cell surface, an order that follows K12 5K (2.8 × 10^6^ nm^2^) < Nissle 1917 (3.3 × 10^6^ nm^2^) ≃ ATCC 25922 (3.4 × 10^6^ nm^2^) < JW4283 (4.5 × 10^6^ nm^2^). Differences in cell size are expected to be coupled to the number of LPS molecules dominating the outer leaflet of the cellular envelope. Indeed, *N*_OS_ follows roughly the order observed for the bacterial outer surface (Table 4 ▸). Normalizing *N*_OS_ values by the bacterial outer surface leads to an LPS surface density of 1.3–1.5 nm^−2^. However, as the cross-sectional area per LPS is ∼1.6 nm^2^ (Clifton *et al.*, 2013 ▸; Micciulla *et al.*, 2019 ▸; Kim *et al.*, 2016 ▸), the expected surface density is ∼0.6 nm^−2^. The discrepancy between the two estimates is most likely due to an underestimation of the bacterial surface by considering the prolate approximation, or uncertainties introduced by β_OS_, which, like *N*_OS_, scales scattering contributions from oligosaccharides and their cross-terms [equation (5)]. An additional factor could be related to the roughness of the bacterial surface (Alves *et al.*, 2010 ▸), which results in a larger effective surface than considered here in our simple estimate.

Finally, the center-to-center distance between the PG layer and the OM Δ_PG_ ≃ 17 nm, with an X-ray SLD ρ_PG_ ≃ 10.2 × 10^−4^ nm^−1^ for all presently studied *E. coli* strains. Previously, we reported Δ_PG_ ≃ 11 nm (Semeraro *et al.*, 2017 ▸), which appears to be more consistent with the length of the lipoproteins cross-linking the peptidoglycan strands to the outer membrane. This deviation from the expected value might be due to the fluctuation modes of Δ_PG_ that are not fully correlated to those of Δ_OM_, which here is modeled by a log-normal distribution function. Devising a separate/partially coupled distribution function for variations of Δ_PG_, is beyond the present experimental resolution, however. In contrast, our new value for 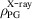 (and also 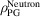 for ATCC) is now consistent with reported hydration values of the peptidoglycan layer, *i.e.* 80–90 vol% (Labischinski *et al.*, 1991 ▸; Pink *et al.*, 2000 ▸). The previously reported value, ∼11.6 × 10^−4^ nm^−1^, included the presence of macromolecular species in the SLD average.

## 7. Conclusion   

The similarity of SAXS data of native and flagellum/fimbria-free *E. coli* strains led us to revise our previously reported scattering-form-factor model (Semeraro *et al.*, 2017 ▸) of the Gram-negative bacterium *E. coli*. The flagellar contribution was replaced by considering the scattering from the oligosaccharide inner and outer cores of the lipopolysaccharides, in terms of a grafted-polymer model. The model presented here is based on detailed compositional and structural estimates of characteristic lengths, volumes and scattering length densities for each cellular component and thus unifies decades of research on *E. coli* ultrastructure and molecular composition into a single comprehensive scattering function. The applicability of the derived model to X-ray and neutron scattering experiments enables the use of the powerful technique of contrast variation in order to highlight or nullify contributions from specific bacterial compartments.

Interestingly, we found that combined (U)SAXS/(V)SANS experiments are not sensitive to the structural heterogeneity of the cytoplasm as the scattering signal of its constituent macromolecules is overwhelmed by the contribution from the cell envelope. Likewise, the combined analysis is not able to report differences in the sub-nanometre range, in particular for cytoplasmic or outer membranes, such as thickness or compositional asymmetry to name but a few. The underlying SLD variations for CM and OM, were therefore fixed at values detailed in Table 2 ▸, along with the width of the peptidoglycan layer and the effective *R*_g_ of each oligosaccharide core. In turn, our technique is highly sensitive to the overall cellular size, the average contrast of the cytoplasmic and periplasmic space, and the structure of the cellular envelope. The last includes the distance between cytoplasmic and outer membranes, as well as its average fluctuations, and the distance between the peptidoglycan layer and the outer membrane. A potential caveat of our model is that the parameters β_OS_, *N*_OS_ and Δ_PG_ can only be determined qualitatively. Specifically, the overall number of LPS molecules (oligosaccaride cores) is affected by approximating the bacterium’s envelope by an ellipsoidal surface, whereas the distance between the peptidoglycan layer and the outer membrane seems to depend on the used intermembrane distance distribution function. Overall, the robustness of our model is demonstrated by an excellent agreement of the derived parameters with a large body of literature on *E. coli* ultrastructure.

In conclusion, elastic scattering experiments on live *E. coli* provide ensemble-averaged values of specific ultrastructural bacterial features without the need of invasive labeling, and are complementary to transmission electron microscopy or optical microscopy. Here we report differences between five *E. coli* strains, which were mainly due to overall size and intermembrane distances (Table 6 ▸). Future research may exploit this platform to detect effects of different sample growth conditions or the effects of bactericidal compounds such as antibiotics. In particular, the combination of our analysis with millisecond time-resolved (U)SAXS enables kinematographic detection of their activity. Our laboratory is currently exploring such an approach for antimicrobial peptides. We also note that devising analogous models for different strains (including Gram-positive bacteria, other simple organisms and cells) requires a similar quality of complementary information to set appropriate physical constraints for the adjustable parameters. Nevertheless, the here-presented model provides ways and guidelines as to how to approach such endeavors.

## 

## Supplementary Material

Supporting information file. DOI: 10.1107/S1600576721000169/vg5126sup1.pdf

URL: https://doi.org/10.5291/ILL-DATA.8-03-910

## Figures and Tables

**Table 1:** Overview of parameters of the revised multi-scale model for live *E. coli* scattering (*cf*. equations 9 and 14)

		Parameters	Description
Cell body	*b*	*n* (ml^−1^)	Cell number density
	*a*	*R* (nm)	Cell radius, centered at the center of mass of the CM
	*b*	ρ_CP_ (nm^−2^)	Average SLD of the cytoplasmic core
	*a*	ɛ	Ratio between major and minor radii

Ultrastructure profile	*a*	*D*_CM_ (nm)	Center-to-center distance between the head-group layers in the CM
	*a*	Δ_OM_ (nm)	Center-to-center distance between CM and OM
	*a*	σ_OM_ (nm)	Standard deviation associated with Δ_OM_
	*a*	*D*_OM_ (nm)	Center-to-center distance between the head-group layers in the OM
	*a*	Δ_PG_ (nm)	Center-to-center distance between the PG layer and the OM
	*a*	*W*_ME_ (nm)	Width of the head-group layers for both CM and OM
	*a*	*W*_PG_ (nm)	Width of the PG layer
	*b*	ρ_TI_ (nm^−2^)	Average SLD of the tail-group layer in the CM
	*b*	ρ_TO_ (nm^−2^)	Average SLD of the tail-group layer in the OM
	*b*	ρ_PP_ (nm^−2^)	Average SLD of the periplasmic layer
	*b*	ρ_ME_ (nm^−2^)	Average SLD of both CM and OM head-group layers
	*b*	ρ_PG_ (nm^−2^)	Average SLD of the PG layer
	*b*	ρ_BF_ (nm^−2^)	SLD of the buffer solution

OS grafting	*a*	*N*_OS_	Number of OS cores, *i.e.* LPS molecules
	*b*	β_OS_ (nm)	β value for each OS core
	*a*	*R*_g,OS_ (nm)	Effective radius of gyration of each OS core

Ribosomes	*c*	*N*_rb_	Number of ribosomes per cell
	*c*	*V*_rb_ (nm^3^)	Volume of a ribosome
	*c*	ρ_rb_ (nm^−2^)	SLD of a ribosome
	*c*	*R*_rb_ (nm)	Radius of the ‘effective’ sphere describing a ribosome

(*a*) Global parameter. (*b*) Local parameter. (*c*) Mixed global and local, but not tested against SANS data. For details, see main text.

**Table 2:** List of fixed parameter values

Parameters	Values
*D*_CM_ (nm)	3.73
*D*_OM_ (nm)	3.33
*W*_ME_ (nm)	0.75
ρ_TI_ × 10^−4^ (nm^−2^)	8.31†/0.022‡
ρ_TO_ × 10^−4^ (nm^−2^)	8.86†/0.012‡
ρ_ME_ × 10^−4^ (nm^−2^)	12.9†/1.24–4.11‡ §
ɛ	2.0/1.75¶
*W*_PG_ (nm)	6.0
*R*_g,OS_ (nm)	0.45

† X-ray SLDs.

‡ Neutron SLDs.

§ These two values, respectively, refer to hydrated head-group SLDs with 0 and 100 wt% D_2_O buffer compositions, accounting for exchangeable H atoms.

¶ ɛ = 2 for ATCC 25922 and ɛ = 1.75 for K12 5K strains.

**Table 3:** Fit results for the global parameters describing USAXS/SAXS and VSANS/SANS of *E. coli* ATCC 25922 strain Errors were calculated from standard deviations of the ensemble of converged fittings. See Table S1 for results on local parameters.

	USAXS/SAXS	VSANS/SANS
*R*	371 ± 3 nm	369 ± 3 nm
ɛ	2.0†	
Δ_OM_ (nm)	34.3 ± 1.0	32.0 ± 1.0
σ_OM_ (nm)	7.4 ± 0.7	7.8 ± 0.3
Δ_PG_ (nm)	17.8 ± 0.2	16.7 ± 1.7
*N*_OS_	(4.7 ± 0.3) × 10^6^	(6.2 ± 0.6) × 10^6^

† Fixed parameter.

**Table 4:** Fitting results for the set of local free parameters for USAXS/SAXS analysis of ATCC 25922, K12 5K, JW4283 and Nissle 1917 strains

Free parameters	ATCC 25922	K12 5K	JW4283	Nissle 1917
*R* (nm)	371 ± 3	363 ± 3	471 ± 4	397 ± 3
ɛ	2.0†	1.75†	1.71 ± 0.03	1.75†
Δ_OM_ (nm)	34.3 ± 1.0	23.8 ± 0.6	26.5 ± 0.8	23.4 ± 0.6
σ_OM_ (nm)	7.4 ± 0.3	4.2 ± 0.2	5.4 ± 0.3	3.7 ± 0.2
Δ_PG_ (nm)	17.8 ± 0.2	16.8 ± 0.2	17.8 ± 0.2	17.3 ± 0.2
*N*_OS_ (×10^6^)	4.7 ± 0.3	4.10 ± 0.12	6.0 ± 0.6	4.09 ± 0.17

† Fixed value.

## References

[bb1] Alves, C. S., Melo, M. N., Franquelim, H. G., Ferre, R., Planas, M., Feliu, L., Bardají, E., Kowalczyk, W., Andreu, D., Santos, N. C., Fernandes, M. X. & Castanho, M. A. R. B. (2010). *J. Biol. Chem.* **285**, 27536–27544. 10.1074/jbc.M110.130955 PMC2934620 20566635

[bb2] Baba, T., Ara, T., Hasegawa, M., Takai, Y., Okumura, Y., Baba, M., Datsenko, K. A., Tomita, M., Wanner, B. L. & Mori, H. (2006). *Mol. Syst. Biol.* **2**, 1–11. 10.1038/msb4100050 PMC1681482 16738554

[bb3] Banzhaf, W., Nordin, P., Keller, R. E. & Francone, F. D. (1998). *Genetic Programming; an Introduction*, Vol. 1. San Francisco: Elsevier.

[bb4] Bennett, B. D., Kimball, E. H., Gao, M., Osterhout, R., Van Dien, S. J. & Rabinowitz, J. D. (2009). *Nat. Chem. Biol.* **5**, 593–599. 10.1038/nchembio.186 PMC2754216 19561621

[bb5] Beveridge, T. J. (1999). *J. Bacteriol.* **181**, 4725–4733. 10.1128/jb.181.16.4725-4733.1999 PMC93954 10438737

[bb6] Breed, R. S. & Dotterrer, W. D. (1916). *J. Bacteriol.* **1**, 321–331. 10.1128/jb.1.3.321-331.1916 PMC378655 16558698

[bb7] Burge, R. E., Fowler, A. G. & Reaveley, D. A. (1977). *J. Mol. Biol.* **117**, 927–953. 10.1016/s0022-2836(77)80006-5 606839

[bb8] Clifton, L. A., Skoda, M. W. A., Daulton, E. L., Hughes, A., Le Brun, A. P., Lakey, J. H. & Holt, S. A. (2013). *J. R. Soc. Interface.* **10**, 20130810. 10.1098/rsif.2013.0810 PMC3808558 24132206

[bb9] Cubitt, R., Schweins, R. & Lindner, P. (2011). *Nucl. Instrum. Methods Phys. Res. A*, **665**, 7–10.

[bb10] De Siervo, A. J. (1969). *J. Bacteriol.* **100**, 1342–1349. 10.1128/jb.100.3.1342-1349.1969 PMC250333 4902814

[bb11] Doi, M. & Edwards, S. F. (1988). *The Theory of Polymer Dynamics*, International Series of Monographs on Physics, Vol. 73. Oxford University Press.

[bb12] Gan, L., Chen, S. & Jensen, G. J. (2008). *Proc. Natl Acad. Sci. USA*, **105**, 18953–18957. 10.1073/pnas.0808035105 PMC2596242 19033194

[bb13] Gundlach, A. R. von, Garamus, V. M., Gorniak, T., Davies, H. A., Reischl, M., Mikut, R., Hilpert, K. & Rosenhahn, A. (2016). *Biochim. Biophys. Acta*, **1858**, 918–925. 10.1016/j.bbamem.2015.12.022 26730877

[bb14] Gundlach, A. R. von, Garamus, V. M., Willey, T. M., Ilavsky, J., Hilpert, K. & Rosenhahn, A. (2016). *J. Appl. Cryst.* **49**, 2210–2216. 10.1107/S1600576716018562 PMC5139998 27980516

[bb15] Guo, A. C., Jewison, T., Wilson, M., Liu, Y., Knox, C., Djoumbou, Y., Lo, P., Mandal, R., Krishnamurthy, R. & Wishart, D. S. (2012). *Nucleic Acids Res.* **41**, D625–D630. 10.1093/nar/gks992 PMC3531117 23109553

[bb16] Heinrichs, D. E., Yethon, J. A. & Whitfield, C. (1998). *Mol. Microbiol.* **30**, 221–232. 10.1046/j.1365-2958.1998.01063.x 9791168

[bb17] Hobot, J. A., Carlemalm, E., Villiger, W. & Kellenberger, E. (1984). *J. Bacteriol.* **160**, 143–152. 10.1128/jb.160.1.143-152.1984 PMC214693 6207168

[bb18] Huxley, H., Faruqi, A., Bordas, J., Koch, M. & Milch, J. (1980). *Nature*, **284**, 140–143. 10.1038/284140a0 7189013

[bb19] Keerl, M., Pedersen, J. S. & Richtering, W. (2009). *J. Am. Chem. Soc.* **131**, 3093–3097. 10.1021/ja807367p 19206229

[bb20] Kim, S., Patel, D. S., Park, S., Slusky, J., Klauda, J. B., Widmalm, G. & Im, W. (2016). *Biophys. J.* **111**, 1750–1760. 10.1016/j.bpj.2016.09.001 PMC5071556 27760361

[bb21] Klein, R. & D’Aguanno, B. (1996). *Light Scattering: Principles and Development*, edited by W. Brown, ch. 2. Oxford: Clarendon Press.

[bb22] Komeda, Y., Kutsukake, K. & Iino, T. (1980). *Genetics*, **94**, 277–290. 10.1093/genetics/94.2.277 PMC1214143 6993282

[bb23] Kučerka, N., Holland, B., Pan, J., Heberle, F. A., Gray, C. G., Tomberli, B. & Katsaras, J. (2012). *Biophys. J.* **102**, 504a–505a.

[bb24] Kučerka, N., van Oosten, B., Pan, J., Heberle, F. A., Harroun, T. A. & Katsaras, J. (2015). *J. Phys. Chem. B*, **119**, 1947–1956. 10.1021/jp511159q 25436970

[bb25] Kučerka, N., Papp-Szabo, E., Nieh, M.-P., Harroun, T. A., Schooling, S. R., Pencer, J., Nicholson, E. A., Beveridge, T. J. & Katsaras, J. (2008). *J. Phys. Chem. B*, **112**, 8057–8062. 10.1021/jp8027963 18549267

[bb26] Labischinski, H., Goodell, E. W., Goodell, A. & Hochberg, M. L. (1991). *J. Bacteriol.* **173**, 751–756. 10.1128/jb.173.2.751-756.1991 PMC207068 1987162

[bb27] Lebedev, D., Paleskava, A., Shvetcov, A., Polyakova, M., Isaev-Ivanov, V. & Konevega, A. L. (2015). Experimental Report LS 2406. ESRF – The European Synchrotron, Grenoble, France.

[bb28] Leber, R., Pachler, M., Kabelka, I., Svoboda, I., Enkoller, D., Vácha, R., Lohner, K. & Pabst, G. (2018). *Biophys. J.* **114**, 1945–1954. 10.1016/j.bpj.2018.03.006 PMC5937145 29694871

[bb29] Liberton, M., Page, L. E., O’Dell, W. B., O’Neill, H., Mamontov, E., Urban, V. S. & Pakrasi, H. B. (2013). *J. Biol. Chem.* **288**, 3632–3640. 10.1074/jbc.M112.416933 PMC3561581 23255600

[bb30] Lieb, M., Weigle, J. J. & Kellenberger, E. (1955). *J. Bacteriol.* **69**, 468–471. 10.1128/jb.69.4.468-471.1955 PMC357561 14367303

[bb31] Lohner, K., Sevcsik, E. & Pabst, G. (2008). *Advances in Planar Lipid Bilayers and Liposomes*, Vol. 6, pp. 103–137. Amsterdam: Elsevier.

[bb32] Maclean, F. I. & Munson, R. J. (1961). *J. Gen. Microbiol.* **25**, 17–27. 10.1099/00221287-25-1-17 13764949

[bb34] Matias, V. R. F., Al-Amoudi, A., Dubochet, J. & Beveridge, T. J. (2003). *J. Bacteriol.* **185**, 6112–6118. 10.1128/JB.185.20.6112-6118.2003 PMC225031 14526023

[bb35] Micciulla, S., Gerelli, Y. & Schneck, E. (2019). *Biophys. J.* **116**, 1259–1269. 10.1016/j.bpj.2019.02.020 PMC6451063 30878200

[bb36] Milne, J. L. S. & Subramaniam, S. (2009). *Nat. Rev. Microbiol.* **7**, 666–675. 10.1038/nrmicro2183 PMC6993139 19668224

[bb37] Müller-Loennies, S., Lindner, B. & Brade, H. (2003). *J. Biol. Chem.* **278**, 34090–34101. 10.1074/jbc.M303985200 12819207

[bb38] Nagy, G., Ünnep, R., Zsiros, O., Tokutsu, R., Takizawa, K., Porcar, L., Moyet, L., Petroutsos, D., Garab, G., Finazzi, G. & Minagawa, J. (2014). *Proc. Natl Acad. Sci. USA*, **111**, 5042–5047. 10.1073/pnas.1322494111 PMC3977285 24639515

[bb39] Narayanan, T., Sztucki, M., Van Vaerenbergh, P., Léonardon, J., Gorini, J., Claustre, L., Sever, F., Morse, J. & Boesecke, P. (2018). *J. Appl. Cryst.* **51**, 1511–1524. 10.1107/S1600576718012748 PMC6276275 30546286

[bb40] Neidhardt, F. C., Ingraham, J. L. & Schaechter, M. (1990). *Physiology of the Bacterial Cell: a Molecular Approach*. Sunderland: Sinauer Associates.

[bb41] Nickels, J. D., Chatterjee, S., Stanley, C. B., Qian, S., Cheng, X., Myles, D. A. A., Standaert, R. F., Elkins, J. G. & Katsaras, J. (2017). *PLoS Biol.* **15**, e2002214. 10.1371/journal.pbio.2002214 PMC5441578 28542493

[bb42] Oursel, D., Loutelier-Bourhis, C., Orange, N., Chevalier, S., Norris, V. & Lange, C. M. (2007). *Rapid Commun. Mass Spectrom.* **21**, 1721–1728. 10.1002/rcm.3013 17477452

[bb43] Pandit, K. R. & Klauda, J. B. (2012). *Biochim. Biophys. Acta*, **1818**, 1205–1210. 10.1016/j.bbamem.2012.01.009 22274566

[bb44] Pedersen, J. S. (1997). *Adv. Colloid Interface Sci.* **70**, 171–210.

[bb45] Pedersen, J. S. (2000). *J. Appl. Cryst.* **33**, 637–640.

[bb46] Pedersen, J. S. & Gerstenberg, M. C. (1996). *Macromolecules*, **29**, 1363–1365.

[bb47] Pink, D., Moeller, J., Quinn, B., Jericho, M. & Beveridge, T. (2000). *J. Bacteriol.* **182**, 5925–5930. 10.1128/jb.182.20.5925-5930.2000 PMC94722 11004199

[bb48] Porod, G. (1982). *Small Angle X-ray Scattering*, edited by O. Glatter & O. Kratky, ch. 2. London: Academic Press.

[bb33] Prasad Maharjan, R. & Ferenci, T. (2003). *Anal. Biochem.* **313**, 145–154. 10.1016/s0003-2697(02)00536-5 12576070

[bb49] Rodriguez-Loureiro, I., Latza, V. M., Fragneto, G. & Schneck, E. (2018). *Biophys. J.* **114**, 1624–1635. 10.1016/j.bpj.2018.02.014 PMC5954297 29642032

[bb50] Schwarz-Linek, J., Arlt, J., Jepson, A., Dawson, A., Vissers, T., Miroli, D., Pilizota, T., Martinez, V. A. & Poon, W. C. K. (2016). *Colloids Surf. B Biointerfaces*, **137**, 2–16. 10.1016/j.colsurfb.2015.07.048 26310235

[bb51] Seltmann, G. & Holst, O. (2002). *The Bacterial Cell Wall*, 1st ed. Berlin, Heidelberg: Springer-Verlag.

[bb52] Semeraro, E. F., Devos, J. M. & Narayanan, T. (2018). *J. Chem. Phys.* **148**, 204905. 10.1063/1.5026778 29865804

[bb53] Semeraro, E. F., Devos, J. M., Porcar, L., Forsyth, V. T. & Narayanan, T. (2017). *IUCrJ*, **4**, 751–757. 10.1107/S2052252517013008 PMC5668860 29123677

[bb54] Semeraro, E. F., Marx, L., Frewein, M. P. K. & Pabst, G. (2020). *Soft Matter*, **17**, 222–232. 10.1039/c9sm02352f 32104874

[bb55] Silhavy, T. J., Berman, M. L. & Enquist, L. W. (1984). *Experiments with Gene Fusions.* Cold Spring Harbor Laboratory.

[bb56] Silhavy, T. J., Kahne, D. & Walker, S. (2010). *Cold Spring Harb. Perspect. Biol.* **2**, a000414. 10.1101/cshperspect.a000414 PMC2857177 20452953

[bb57] Sonnenborn, U. (2016). *FEMS Microbiol. Lett.* **363**, Fnw212. 10.1093/femsle/fnw212 27619890

[bb58] Stukalov, O., Korenevsky, A., Beveridge, T. J. & Dutcher, J. R. (2008). *Appl. Environ. Microbiol.* **74**, 5457–5465. 10.1128/AEM.02075-07 PMC2546619 18606791

[bb59] Turner, L., Stern, A. S. & Berg, H. C. (2012). *J. Bacteriol.* **194**, 2437–2442. 10.1128/JB.06735-11 PMC3347194 22447900

[bb60] Tweeddale, H., Notley-McRobb, L. & Ferenci, T. (1998). *J. Bacteriol.* **180**, 5109–5116. 10.1128/jb.180.19.5109-5116.1998 PMC107546 9748443

[bb61] Whitfield, C. & Roberts, I. S. (1999). *Mol. Microbiol.* **31**, 1307–1319. 10.1046/j.1365-2958.1999.01276.x 10200953

[bb62] Yamashita, I., Hasegawa, K., Suzuki, H., Vonderviszt, F., Mimori-Kiyosue, Y. & Namba, K. (1998). *Nat. Struct. Biol.* **5**, 125–132. 10.1038/nsb0298-125 9461078

[bb63] Zemb, T. & Lindner, P. (2002). *Neutrons, X-rays and Light: Scattering Methods Applied to Soft Condensed Matter.* North-Holland Delta Series. Amsterdam: Elsevier.

[bb64] Zimmerman, S. B. & Trach, S. O. (1991). *J. Mol. Biol.* **222**, 599–620. 10.1016/0022-2836(91)90499-v 1748995

